# Unification Close at Hand

**Published:** 1903-11

**Authors:** 


*EDITOR:*

WILLIAM WARREN POTTER, M. D.

All communications, whether of a literary or business nature, books for review and exchanges should be addressed to the editor: 284 FRANKLIN STREET, BUFFALO, N. Y.

VOL. XLIII.—LIX. NOVEMBER, 1903. No. 4.

### Unification Close at Hand.

THE Medical Society of the State of New York took initiative action, relating to medical unity at its annual meeting, held at Albany in January, 1902, by authorising the appointment of a committee of conference, in accordance with the recommendation of President Elsner in his inaugural address, the part of which pertaining to that subject, reading as follows:

That the Medical Society of the State of New York appoint a committee of five, to confer with an equal number representing the New York State Medical Association, for the purpose of formulating a plan which shall have for its object the reorganisation of the regular profession of this state, which body shall be in affiliation with the American Medical Association, and that the committee report the result of its labors at the next meeting of the Medical Society of the State of New York. In the event of failure of the New York Medical Association to appoint such committee, or if the committee should fail to agree upon a plan of reorganisation, the committee appointed by the Medical Society of the State of New York shall have full power, if it deems it expedient, to represent this society before the American Medical Association, and the secretary of this society shall, if the majority of the committee desire, provide the individual members with credentials to represent this society in the American Medical Association.

The committee thus created pursued its labors during the year 1902, in conference with a similar committee of the New York State Medical Association, and at the annual meeting of the Medical Society of the State of New York, held in January, 1903, reported the results of its labors in detail to the latter society. The report concluded as follows:

In reporting progress your committee wishes:

1. To file its objection to any method of reorganisation which shall in any way interfere with the life of the Medical Society of the State of New York. Therefore, we are opposed to any legislative act in connection with the amalgamation of the medical profession of the State of New York. The opinion of Judge Andrews makes it clear that no such action is necessary.

2. Your committee having been assured that the old code of ethics of the American Medical Association is still in existence, does not feel that it is within its power to recommend that action be taken by the Medical Society of the State of New York, until the American Medical Association shall make it possible for the Medical Society of the State of New York to subscribe to its constitution and by-laws and written rules of order, consistently, without the sacrifice of principle.

3. The methods of gathering data which shall serve to formulate a plan of organisation ultimately to be presented to this body for approval, must be evolved after further conference and greater consideration than your committee has as yet been able to give to this important subject.

4. The Medical Society of the State of New York cannot continue to issue the Directory and the monthly Journal, now being published by the New York State Medical Association, in the event of reorganisation.

For the foregoing reasons the committee asked that its report be received and that further time be given it for the development of the purposes for which it was created.

Soon after the American Medical Association abrogated its code of ethics in May last, the chairman of the committee invited further conference with the New York State Medical Association, and on October 1, 1903, the latter body appointed a new committee, conferring upon it full power to effect a union of the two organisations as will be seen by the following resolutions, offered by Dr. Joseph D. Bryant and seconded by Drs. John A. Wyeth and E. D. Ferguson, which were passed unanimously:

WHEREAS, The members of The New York State Medical Association desire a union of the medical profession in the State of New York; and

WHEREAS, It is deemed expedient for the attainment of this purpose to make further effort to bring together The New York State Medical Association and the Medical Society of the State of New York under the name of "The Medical Society of the State of New York";

*Resolved,* That a committee of five be appointed by the chair, and said committee is hereby empowered to do whatever is necessary and expedient to bring about such a union in a just and equitable manner; and

*Resolved,* That the committee so empowered may confer, cooperate and unite with a committee of the Medical Society of the State of New York for the purpose of forming said union of the two medical organisations; and

*Resolved,* That a copy of these resolutions be transmitted to the secretary of the Medical Society of the State of New York, with a request that their Conference Committee be granted similar powers.

The following named committee was appointed in accordance with the above resolutions: E. Eliot Harris, chairman; Julius C. Bierwirth, Alexander Lambert, Parker Syms and Wisner R. Townsend.

At a special meeting of the Medical Society of the State of New York, held in the New York Academy of Medicine at New York City, Tuesday, October 13, 1903, the president, Dr. A. T. Bristow, of Brooklyn, stated the object of the meeting in well-chosen words. Dr. D. B. St. John Roosa, of New York, then rose and said:

As one of those who have lived long enough to see the beginning and, I hope, will live long enough to see the ending of this controversy that has been carried on so long, I have the pleasure of offering the following resolution, which, I think, will have the vote of every member of the Medical Society of the State of New York.

He then presented the preamble and resolution which read as follows:

Whereas, The New York Medical Association, at a recent special meeting duly assembled, has, by unanimous vote, appointed a committee, with full power, to meet with the similar committee of the Medical Society of the State of New York to arrange for the unification of the two organisations under the corporate name of the Medical Society of the State of New York; therefore, be it

*Resolved,* That the Committee of Conference of the Medical Society of the State of New York, already appointed, be given power equal to and commensurate with the powers recently granted the committee created by the New York State Medical Association for the purpose of unifying the two state medical societies into the Medical Society of the State of New York.

Dr. Willis G. Macdonald, of Albany, seconded the motion of Dr. Roosa in a brief speech, remarkable for its appropriate phraseology and good form. Both gentlemen received the generous applause of the large house and a unanimous vote of adoption completed the session, which immediately adjourned. (See p. 256.)

By this action of the society the committee representing it is clothed with ample authority to adjust all questions relating to union that are likely to arise. Its personnel is such as to inspire implicit confidence in its action. As will be remembered, it is composed as follows: Henry L. Elsner, chairman; A. Jacobi, A. Vander Veer, George Ryerson Fowler, and Frank Van Fleet. By the time appointed for the Medical Society of the State of New York to hold its ninety-eighth annual meeting at Albany, January 26-28, 1904, we confidently predict that the unification of the medical profession in the Empire State will be an accomplished fact.

